# Acknowledgement to Reviewers of *Behavioral Sciences* in 2013

**DOI:** 10.3390/bs4010070

**Published:** 2014-02-26

**Authors:** Behavioral Sciences Editorial Office

**Affiliations:** MDPI AG, Klybeckstrasse 64, CH-4057 Basel, Switzerland

The editors of *Behavioral Sciences* would like to express their sincere gratitude to the following reviewers for assessing manuscripts in 2013:

Alho, Päivi
Allen, Andrew
Aretouli, Eleni
Beebe, John
Blasche, G.
Burnham, John C.
Butler, David L.
C'de Baca, Janet
Caillouet, Beth
Cambray, Joseph
Cardo, Esther
Carrera, Pilar
Chang, Luke J.
Chee, Christine
Clair, Michelle C. St
Clapper, John
Clay, Andrea W.
Craddock, Travis
Danckert, James
Darlington, Beth
Dawson, Terence
De la Torre, Gabriel G.
Deshmukh, Vinod
Devonis, David C.
Diestel, Stefan
Eddington, Kari M.
Elliot, Andrew
Elmore, Lauren Caitlin
Fidalgo, C.
Fleming, Jennifer
Frasca, Diana
Frowd, Charlie D.
Gawęda, Łukasz
Gilbert, Avery
Guo, Gao
Harasymchuk, Cheryl
Hartley, James
Hashimoto, Ryu-Ichiro
Hinton, W. Ladson
Hirvonen, Riikka
Hodgson, Tim
Hoff, Robert
Ishitobi, Makoto
Jasper, Fabian
Jimeno, Natalia
Kak, Subhash
Kamens, Helen
Keller, Andreas
Kitamura, Christine
Krishnan, Mohan
Lane, Andrew
Lany, Jill
Li, Xia
Lienard, Pierre
Maack, Danielle
Macdonald, Kevin
Mackay, Harry A.
Maclean, Evan L.
Main, Roderick
Martyr, Anthony
Matthews, Gerald
Mcgann, Marek
Mills, Jon
Morimura, Naruki
Morioka, Masayoshi
Muse, Mark
Näätänen, Risto
Nadler, Ruby T.
Niznikiewicz, Margaret
Nolan, Steve
Nowicki, Steve
Oblak, Adrian L.
Oertel, Viola
Oostenbroek, Janine
Pauly, Katharina
Peet, Christopher
Petrazzini, Maria Elena Miletto
Ploeger, Annemie
Price, Katy
Ragland, Daniel
Ravizza, Susan M.
Reardon, Annemarie
Reidy, Dennis E.
Roesler, Christian
Rosegrant, John
Saban, Mark
Saksida, Lisa M.
Schipul, Sarah E.
Schmitter-Edgecombe, Maureen
Schott, Björn H.
Segal, Robert
Shakeel, Mohammed Kalathil
Sharp, Carla
Sheehy, Maura
Smythe, William
Snowden, Charles
Sparrow, G. Scott
Stein, Murray
Stone, Ws
Storm, Lance
Susswein, Noah
Szpunar, Karl
Talpos, John
Topolovec-Vranic, Jane
Turner, Robert
Udal, Anne H
Uher, Jana
Van Der Lugt, Arie
Van Tilburg, Wijnand A. P.
Walach, Harald
Walkerdine, Valerie
Warburton, E.C.
Weishaus, Joel
Williams, Lisa
Zoladz, Phillip R.

